# From fragmented to integrated surveillance in LMICs: digital pathways for outbreak detection and vaccine intelligence

**DOI:** 10.3389/fpubh.2026.1843782

**Published:** 2026-08-07

**Authors:** Delfin Lovelina Francis, Saravanan Sampoornam Pape Reddy

**Affiliations:** 1 Saveetha Dental College & Hospitals, Saveetha University, SIMATS, Chennai, India; 2 Department of Periodontology, Army Dental Corps, New Delhi, India

**Keywords:** artificial intelligence, digital health, immunization programs, infectious disease surveillance, interoperability, low- and middle-income countries, one health, public health informatics

## Abstract

**Background:**

Low- and middle-income countries (LMICs) often rely on fragmented, disease-specific surveillance systems that produce delayed and incomplete data. Recent outbreaks, including COVID-19, have highlighted the urgent need for integrated, real-time digital surveillance to improve early outbreak detection and optimize vaccine deployment.

**Methods:**

A structured narrative review was conducted following SANRA recommendations on the literature published between 2019 and 2025 on digital health, artificial intelligence (AI), genomic surveillance, and vaccine-information systems in LMIC infectious disease surveillance. PubMed, Scopus, WHO IRIS, and regional CDC repositories were searched using pre-specified Boolean strings. Over 312 records were screened; 46 unique references were selected for final synthesis.

**Results:**

Traditional indicator-based systems in LMICs suffer from siloed reporting, poor connectivity, and workforce shortages. Digital platforms (DHIS2, mobile reporting, cloud dashboards) can unify multi-sector data and accelerate outbreak signals. AI tools offer predictive capabilities, though LMIC-specific external validation remains limited in only 14% of published models. Electronic immunization registries demonstrate measurable improvements: stockout reductions of up to 76%, coverage gains of 12.3%, and median reporting delays cut from 28 to 3 days. Key barriers include non-standardized data formats, intermittent connectivity, algorithmic bias, and data governance gaps.

**Conclusions:**

Achieving integrated surveillance in LMICs requires adherence to interoperability standards, robust digital infrastructure, One Health data integration, and equitable data governance. A four-layer conceptual framework is presented. Investment in local capacity, supportive policy, and ethical AI frameworks is critical for sustainable innovation.

## 1 Introduction

Infectious disease surveillance is the basis of epidemiology, which is envisioned to promptly identify outbreaks and direct public health interventions (1). Global initiatives, such as the International Health Regulations and the Integrated Disease Surveillance and Response (IDSR) WHO framework, have encouraged integrated, multi-disease surveillance; however, data is still fragmented and siloed in many LMICs (2, 3). Vertical programs for polio, tuberculosis, and malaria sometimes gather data in parallel without interoperable exchange, resulting in redundant work and blind spots in surveillance (4). Manual reporting from clinics and labs is the foundation of traditional indicator-based systems, which causes delays and insufficient coverage (5). These structural flaws were brought to light by the COVID-19 pandemic, which significantly hampered responses due to delayed laboratory confirmations and reliance on paper records highlighting the urgent need for digital transformation in surveillance.

AI and digital health are viable solutions to fragmentation. Clinical case reports, genetic sequences, movement data, and social media feeds are just a few of the streams that big data and AI may combine to enable early epidemic warning and precise targeting (6, 7). In order to improve vaccine delivery equity, digital vaccine intelligence solutions, such as electronic stock management platforms and immunization registries, can provide real-time tracking of coverage and cold-chain logistics (8, 9). To enable proactive, coordinated responses, a single epidemic intelligence platform could theoretically include immunization dashboards, genetic variation tracking, and syndromic alerts.

This structured narrative review synthesizes contemporary research on digital surveillance in LMICs, emphasizing interoperability, AI-driven outbreak detection, genomic surveillance, and digital immunization systems. Barriers and success factors are identified from recent studies and reports, guided by the SANRA recommendations for structured narrative reviews (10). The aim is to outline how LMICs can evolve from fragmented data systems to integrated, collaborative surveillance for better outbreak detection and vaccine decision-making.

## 2 Methods

### 2.1 Review design and SANRA compliance

The study was conducted as a structured narrative review following the Scale for the Assessment of Narrative Review Articles (SANRA), which provides methodological guidance for transparent literature identification, critical appraisal, and balanced evidence synthesis. Because the objective was to provide a conceptually integrated overview rather than an exhaustive evidence map, the review was not designed as a systematic review or a formal scoping review. Nevertheless, selected principles from the PRISMA Extension for Scoping Reviews (PRISMA-ScR) were used solely to improve the transparency of literature searching, study selection, and reporting. No meta-analysis or formal evidence mapping was undertaken. (11).

### 2.2 Inclusion and exclusion criteria

Inclusion criteria: (1) peer-reviewed articles, gray literature, and official reports published between January 2019 and December 2025; (2) studies conducted in or directly applicable to LMICs (World Bank classification); (3) studies addressing at least one domain; digital disease surveillance, AI-driven outbreak detection, genomic epidemiology, electronic immunization registries, or One Health data integration; (4) English-language publications; (5) quantitative, qualitative, or mixed-methods designs. Exclusion criteria: (1) studies exclusively focused on high-income country settings without LMIC applicability; (2) pre-2019 publications unless seminal (cited in ≥50 reviews); (3) opinion pieces lacking empirical data; (4) conference abstracts without full text; (5) studies restricted to non-communicable disease surveillance.

### 2.3 Search strategy

Databases (PubMed, Scopus) and institutional repositories (WHO IRIS, Africa CDC, PAHO) were queried. The primary PubMed string was: (“digital surveillance” OR “electronic surveillance” OR “integrated surveillance”) AND (“LMIC” OR “low-income country” OR “middle-income country” OR “sub-Saharan Africa” OR “South Asia”) AND (“artificial intelligence” OR “machine learning” OR “genomic surveillance” OR “vaccine registry” OR “immunization information system” OR “outbreak detection” OR “One Health”). Equivalent strings were adapted for each additional source (full strings: Supplementary Appendix A).

### 2.4 Selection process (reported using PRISMA-ScR principles)

Records identified through database searches (*n* = 312) were screened after duplicate removal (*n* = 268). Following title and abstract screening, 118 records were excluded, and 150 full-text articles were assessed for eligibility of these, 100 articles were excluded based on the predefined eligibility criteria, resulting in 46 references included in the final narrative synthesis.

### 2.5 Critical appraisal and evidence grading

Included studies were assessed along three dimensions: (1) Evidence strength: Strong (multi-country prospective or controlled studies; systematic reviews), Moderate (observational cohort; single-country evaluations), or Weak (case reports; gray literature); (2) Risk of bias: selection bias, information bias, and algorithmic bias (AI models trained on non-LMIC populations); and (3) Generalizability across LMIC sub-regions (Supplementary Table S1).

## 3 Fragmented vs. integrated surveillance systems

LMIC surveillance frequently remains extremely fragmented. Siloed reporting: Many countries function parallel disease programs and separate systems for malaria, cholera, and measles that do not share data (2, 4). Polio digital reporting system of Nigeria improved timeliness but suffered incompleteness due to poor internet connectivity and lack of unified standards; Uganda's mobile TB reporting enhanced real-time alerts but failed to link with laboratory databases, resulting in lost confirmations (5). Infrastructure gaps: Unstable power, low network coverage, and reliance on paper registers hamper many LMICs (5, 6). Workforce constraints: Many health systems lack trained informaticians and data clerks; health workers in Sierra Leone, India, and elsewhere report being overloaded by multiple reporting tools.^2^ In sum, existing systems are often “weak, siloed and under-resourced,” leading to under-reporting and delayed outbreak signals (1, 2).

**Figure 1:**
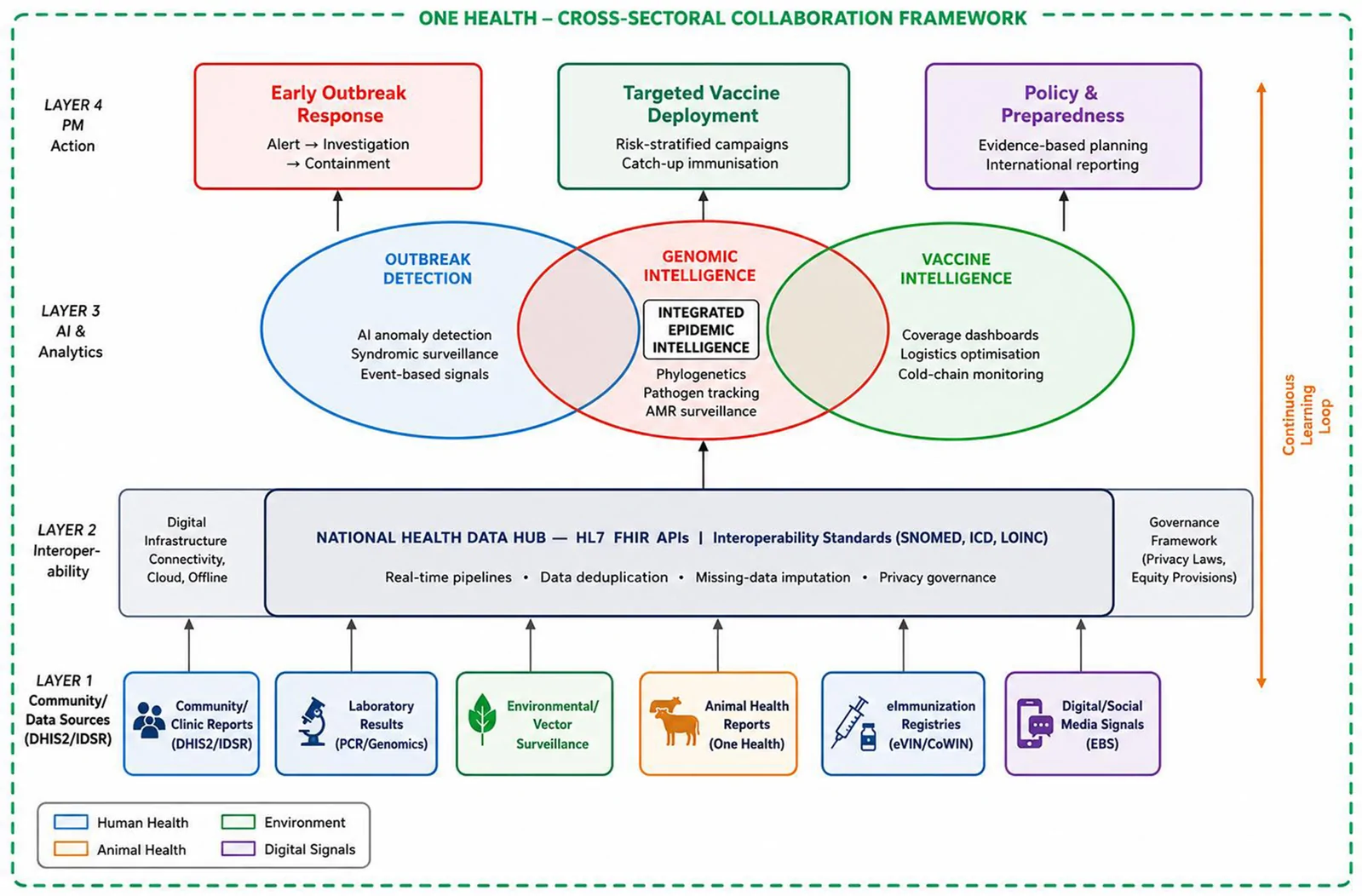
Conceptual framework: from fragmented to integrated surveillance in LMICs. Conceptual framework illustrating the transition from fragmented surveillance systems to integrated epidemic intelligence in LMICs. The framework depicts four interconnected layers comprising multisectoral data inputs, an interoperable National Health Data Hub, an AI-enabled decision-support engine integrating outbreak detection, genomic surveillance, and digital vaccine intelligence, and evidence-informed public health actions operating within a continuous learning cycle. The framework was developed by the authors based on synthesis of published literature (3, 6–8, 13–15).

By contrast, integrated digital systems bring all data under one platform. Shared electronic health information systems such as DHIS2 can collect case data for multiple diseases, feeding into dashboards and analytics. Moderate evidence from a prospective pre–post evaluation involving 47 Kenyan health facilities demonstrated that SMS-based reporting reduced the median reporting delay by approximately 60% (28 to 11 days). Although promising, these findings originated from a programmatic implementation and require validation across broader LMIC settings (12). Cloud-based databases and geospatial mapping tools enable immediate visualization of case clusters. Advances in mobile devices, GIS, and machine-learning analysis can detect anomalies that would escape traditional systems (1, 7). Table 1 systematizes key contrasts between fragmented and integrated approaches. Figure 1 presents the conceptual framework for this transition.

**Table 1:** Surveillance approaches: fragmented vs. integrated.

Feature	Fragmented (traditional)	Integrated digital systems
Data collection	Vertical disease-specific programs; paper-based; manual aggregation	Unified HIS (e.g., DHIS2); electronic case reporting; mobile apps (2, 6)
Data flow	Disconnected silos; delayed batch reporting	Interoperable databases; real-time pipelines using HL7 FHIR (6)
Outbreak detection	Delayed lab-confirmed reports; low sensitivity	AI-driven anomaly detection; syndromic and event-based surveillance (7)
Vaccine information	Paper stock registers; manual tracking	eVaccine management (eVIN/CoWIN) with real-time stock and coverage dashboards (13)
Stakeholder engagement	Sectoral (human health only)	Multi-sectoral one health—human, animal, and environmental health (14)

Comparison of traditional fragmented surveillance systems and integrated digital surveillance architectures in LMICs, highlighting differences in data collection, interoperability, outbreak detection, vaccine information management, and multisectoral collaboration. Source: Developed by the authors based on synthesis of published literature (2, 5–7, 13–15).

## 4 Digital interoperability and architecture

Interoperability, or the capacity of systems to effortlessly share and use information, is a requirement for integration. The lack of common standards in many LMIC health information systems leads to separate databases. Adoption of international data standards (HL7 FHIR for clinical data, SNOMED/ICD-10 for coding) and national interoperability frameworks are important facilitators, according to a recent scoping analysis that revealed LMICs often struggle with old systems and deficiencies in digital infrastructure (6). The industry standard for health data exchange is H7′s Fast Healthcare Interoperability Resources (FHIR), which enables smooth data sharing between various applications. The WHO Global Strategy on Digital Health (2020–2025) explicitly advocates open standards and shared data formats (15).

Despite these advances, governance and investment lags persist. Adekoya et al noted that LMICs often lack the policy, infrastructure, and trained workforce to implement interoperability equitably. Multi-stakeholder data governance bodies and public–private partnerships are essential to ensure that small clinics can connect to national networks without data ownership conflicts (6).

## 5 AI and big data for outbreak detection

AI and big data analytics are transforming outbreak detection, part of a broader shift toward AI-enabled diagnostics, treatment planning, and operational efficiency across health systems (16). Drawing from more than 100 data sources in 65 languages, BlueDot used NLP and airline data to warn of COVID-9′s spread 9 days prior to official WHO alerts (17). EpiTweetr from the ECDC searches social media in several languages for signs of illness. By recording global signals in real time, these “digital epidemiology” technologies supplement conventional indicator-based surveillance. However, there are particular difficulties with AI use in LMIC monitoring. Unreliable connectivity restricts continuous data streams, and legacy data incompatibilities hinder model training. Adoption is slowed by algorithmic bias, which occurs when models are trained mostly on high-income nation epidemic curves (18). As demonstrated by Google Flu Trends, which consistently overestimated influenza rates owing to search query confounding, AI systems produce a large number of alerts that need human validation (19).

### 5.1 Comparative critique of AI surveillance systems

A critical comparison reveals important performance differences. Significant performance disparities are shown by a critical comparison. Although BlueDot's proprietary architecture and reliance on aviation data hinder replication in LMICs with poor flight connectivity, BlueDot (event-based NLP with flight data) demonstrated a 9-day advance warning over WHO for COVID-19. In multi-lingual LMIC validation, EpiTweetr (open-source social media monitoring) demonstrated sensitivity of just 38%−52% with substantial false-positive rates (20). Moderate evidence, primarily derived from retrospective validation studies, suggests that PandemicLLM/EpiLLM systems achieve sensitivities of approximately 71% and specificities of 63%. However, because prospective LMIC validation remains limited, these findings should be interpreted as proof-of-concept rather than evidence of operational readiness (21). In Rwanda, outbreak reporting delays were reduced by 42% (from median 21 to 12 days; Moderate evidence) thanks to DHIS2-integrated ML anomaly detection modules (22). Positive predictive values between 20 and 45% across systems entail verification loads that may be too much for health systems with limited resources (23). Strong evidence from recent reviews indicates that only approximately 14% of published AI surveillance models have undergone external validation in LMIC settings, highlighting a substantial implementation gap and limiting confidence in widespread deployment (24).

Although retrospective validation studies have demonstrated encouraging predictive performance, these findings should primarily be regarded as proof-of-concept rather than evidence of operational readiness. Most AI surveillance models have not undergone prospective evaluation under routine LMIC programme conditions, where data quality, reporting completeness, infrastructure limitations, and epidemiological heterogeneity may substantially influence model performance. Consequently, AI-enabled surveillance should be implemented as a decision-support technology that complements existing epidemiological surveillance rather than as an autonomous outbreak detection system. Human-in-the-loop validation remains essential to verify alerts, minimize false positives, and ensure that algorithmic outputs are interpreted within the local epidemiological context.

### 5.2 AI surveillance pipeline for LMICs

Five successive levels make up an efficient AI surveillance pipeline for LMICs (see Figure 1, Layer 3): (1) DHIS2 case reports, laboratory HL7 FHIR feeds, community health worker mobile apps (offline-first), environmental sensors, and social media APIs; (2) Pre-processing and Standardization automated deduplication, ICD-10 normalization, and missing-data imputation; (3) Feature Extraction spatial clustering (SaTScan), temporal anomaly scoring, and genomic variant classification; (4) Model Layer ensemble gradient boosting for structured data and fine-tuned LLMs for unstructured signals, with calibrated probability outputs; and (5) The final stage incorporates human-in-the-loop validation, whereby epidemiologists and public health authorities review AI-generated alerts before public health action is initiated. This oversight allows contextual interpretation of model outputs, minimizes verification burden arising from false-positive alerts, and ensures that surveillance decisions remain consistent with local epidemiological priorities and governance requirements.

## 6 Genomic surveillance and one health integration

Advances in pathogen genomics have become a vital component of modern surveillance. Sequencing viruses or bacteria yields data to track transmission chains and detect variants. Next-generation sequencing (NGS) was quickly integrated into SARS-CoV-2 monitoring in high-income countries; LMICs are quickly catching up through programs like the Africa CDC's Pathogen Genomics Initiative (25). Before COVID-19, only about 39% of low-income countries had shared influenza genome data with international databases, compared to 78% of wealthier countries; only five countries in Africa accounted for roughly 75% of sequencing capability (26). In LMICs, genomic programs frequently started out as vertical studies (for cholera or Ebola), creating data silos where analysis are carried out overseas without connecting sequences to case surveillance databases (27). Unprecedented exchange of SARS-CoV-2 genomes from LMICs is possible, as the COVID-19 experience showed (28). To motivate local scientists to submit data, strong data-sharing agreements with fair benefit-sharing clauses are crucial (27).

### 6.1 One health integration model

A functional One Health integration model for LMICs comprises three converging data spheres within a shared governance framework. Community health worker reports, facility DHIS2 case records, laboratory genetic sequences, and electronic immunization registration data are all included in the Human Health Sphere. Veterinary laboratory results, wildlife monitoring data, and livestock morbidity and mortality reports (compatible with FAO EMPRES-i) are all included in the Animal Health Sphere. The Environmental Sphere comprises vector surveillance, water quality monitoring, rainfall and temperature anomaly feeds, and land-use change indicators collected from satellites.

A tripartite committee oversees the National One Health Data Hub, where these three domains come together to compute composite spillover risk indicators using standardized ontologies (SNOMED, LOINC, and OIE codes) (29). Joint human-animal monitoring programs identified zoonotic epidemics a median of 18 days earlier than human-only systems, according to a 2023 multi-country East African evaluation (Moderate evidence) (30). However, fewer than 10% of LMICs currently have operational One Health data platforms, and most efforts remain project-based. The Bangladesh Nipah case illustrates the consequences: fruit bat population data were not linked to health data, delaying warnings (31).

## 7 Vaccine intelligence and digital immunization systems

Applying data analytics to immunization systems is becoming more and more important, going beyond outbreak detection and vaccine intelligence. In India, the electronic Vaccine Intelligence Network (eVIN) optimizes distribution by using mobile devices to track cold-chain temperature and stock levels. Strong evidence, based on national programme evaluations of India's Electronic Vaccine Intelligence Network (eVIN), demonstrated stockout reductions of up to 76% together with substantial improvements in cold-chain compliance (32).

Digital vaccination certificates, appointment scheduling, and individual vaccine usage tracking are all provided by India's CoWIN platform. Moderate evidence from an observational evaluation of Tanzania's Electronic Immunization Registry demonstrated a 12.3 percentage-point increase in vaccine coverage. As this finding originated from routine implementation settings, residual selection bias cannot be excluded (33).

Beyond improving data collection and logistics, digital vaccine intelligence systems increasingly support operational public health decision-making. Persistent vaccine hesitancy remains a major driver of under-immunization in both LMIC and high-income settings, reinforcing the value of digital intelligence systems capable of flagging hesitancy-related coverage gaps in near real time (34). Importantly, these systems facilitate monitoring of vaccine uptake among high-risk population subgroups—for example, patients with chronic disease, who show measurably lower coverage than the general population—rather than relying solely on aggregate national coverage indicators, thereby supporting more equitable and precision-oriented immunization strategies (35). Complementing digital vaccination records with serological surveillance can further identify hidden immunity gaps and guide evidence-based vaccination policies, particularly in populations where administrative coverage estimates may not accurately reflect population immunity, as demonstrated by seroprevalence-based approaches used to detect residual immunity gaps against vaccine-preventable diseases such as poliovirus (36). These capabilities strengthen the role of vaccine intelligence as an essential component of integrated epidemic intelligence rather than a standalone logistics management system.

### 7.1 Quantitative evidence summary

Key published metrics from included studies: (1) Strong evidence from national programme evaluations demonstrated stockout reductions of up to 76%.; cold-chain compliance 72% → 93%, *n* = 680 sites (Strong); (2) Moderate evidence from a Tanzanian observational evaluation demonstrated a 12.3 percentage-point increase.; (3) Kenya SMS reporting median delay 28 → 3 days (Strong; prospective comparison, 47 facilities); (4) A district-level pilot evaluation in Bangladesh reported a 34% reduction in vaccination dropouts following implementation of an electronic immunization registry. These findings provide encouraging implementation evidence but require confirmation in larger-scale national programmes (Moderate evidence from a district-level implementation study demonstrated a 34% reduction in loss to follow-up); (5) A single-site pilot implementation in Nigeria demonstrated a reduction in cold-chain breach response time from 6.2 to 1.1 h. Given the limited scale of this evaluation, the findings should be regarded as preliminary and interpreted cautiously until validated in broader operational settings (33, 37). (Supplementary Table S2).

Collectively, these findings demonstrate the potential value of digital surveillance and vaccine intelligence platforms. However, many estimates originate from pilot implementations, national flagship programmes, or observational evaluations conducted under favorable implementation conditions. Consequently, direct extrapolation to routine LMIC health systems should be undertaken cautiously until additional multicountry prospective evaluations become available (Table 2).

**Table 2:** Vaccine programme challenges vs. digital solutions.

Challenge (immunization)	Traditional data system	Digital vaccine intelligence (EIR/eVIN/CoWIN)
Manual stock management	Paper logs; often inaccurate	Real-time electronic inventory; stockout alerts (13)
Lost vaccination records	Physical cards; poor retrieval	Centralized digital certificates nationwide (8)
No real-time coverage data	Delayed aggregate reports	Instant dashboards of coverage, dropouts, adverse events (8)
Cold chain monitoring	Manual logs; lag in response	Sensor-enabled temperature feeds; rapid alerts (33)
Follow-up of beneficiaries	Manual planning; no outreach	Automated SMS reminders and defaulter tracking (8)
Inclusion of mobile populations	Often excluded (lost cards)	Portable records via unique health IDs (8)
Data for decision-making	Fragmented spreadsheets	Integrated analyses identifying high-risk clusters (9)

Comparison of conventional immunization information systems and digital vaccine intelligence platforms illustrating operational challenges, digital solutions, and corresponding public health functions. Source: Developed by the authors based on synthesis of published literature (8, 9, 13, 31, 32, 37).

## 8 Barriers and enablers of implementation

### 8.1 Technical infrastructure

There is limited and inconsistent internet connectivity in many areas. Offline-first software and labs powered by solar or batteries are partial solutions, but they cost money. Systems are additionally burdened by the shift from analog to digital.

### 8.2 Human capacity

There is a shortage of qualified data managers and analysts. Data gaps result from health workers' frequent difficulties with new programs, according to surveys conducted in Africa. Workforce constraints can be addressed by providing community health workers with ongoing education through task-shifting and tele-mentoring.

### 8.3 Governance and policy

Initiatives supported by donors occasionally establish rival reporting systems. Digital sharing is discouraged in several nations due to the absence of data privacy laws. Concerns about “data colonialism”, the practice of outside parties extracting value without fairly sharing benefits call for collaboration with regional scientists and open data practices (38).

### 8.4 Financial resources

Consistent funding is essential. Multi-donor projects frequently concentrate on vertical outcomes; nations must set aside funds for data centers, analytics teams, and IT infrastructure upkeep. Costs can be reduced through public-private partnerships. The following are important facilitators: (1) international collaboration, such as the Africa CDC's Pathogen Genomics Initiative, provides models of regional cooperation; (2) open-source tools, such as DHIS2, KoBoToolbox, and OpenMRS, lower costs and allow customization; (3) mobile penetration, high smartphone adoption creates opportunities for offline-first tools; (4) global standards, such as WHO's Digital Health Interoperability Framework, facilitate future integration; and (5) post-COVID-19 political momentum, where many governments recognize the ROI of early detection (39).

Beyond improving outbreak detection, integrated digital surveillance contributes to broader health-system resilience by enabling adaptive responses during and after major infectious disease emergencies. The COVID-19 pandemic demonstrated that fragmented reporting systems, delayed laboratory confirmation, and reliance on paper-based information systems not only impeded epidemic control but also disrupted routine healthcare delivery and resource allocation. Recent assessments of the evolving post-pandemic epidemiological landscape reinforce this point, highlighting that surveillance, misinformation management, and preparedness planning remain critical even as the acute phase of COVID-19 recedes (40). Integrated surveillance platforms can strengthen these adaptive capacities by improving situational awareness, facilitating dynamic prioritization of limited resources, and supporting evidence-informed decision-making across multiple levels of the health system.

Governance and equity are fundamental to the successful implementation of integrated digital surveillance systems. While concerns regarding data ownership, privacy protection, algorithmic bias, and data colonialism remain important, effective implementation also requires robust governance safeguards. National data governance bodies should establish clear regulatory frameworks governing data stewardship, interoperability, ethical AI deployment, and cross-sector data sharing. AI models should undergo local validation before operational use to ensure acceptable performance across diverse epidemiological and demographic settings. Adoption of open interoperability standards and transparent procurement processes can reduce vendor lock-in and improve long-term sustainability. Equitable benefit-sharing arrangements should ensure that countries contributing surveillance and genomic data receive appropriate scientific, technical, and public health benefits. Strong cybersecurity measures, role-based access controls, audit trails, and compliance with national data protection legislation are essential to safeguard sensitive health information. Finally, maintaining community trust through transparency, stakeholder engagement, and clear accountability mechanisms for false-positive alerts, missed outbreak signals, and algorithmic errors will be critical for achieving sustainable implementation of integrated epidemic intelligence platforms in LMICs.

## 9 Case studies of integrated surveillance

### 9.1 India (infectious disease and immunization)

India's Ayushman Bharat Health Account links CoWIN and eVIN data, representing a pathway toward unified health records. Rural health workers use Android-based apps that work offline and sync with the national server, bridging connectivity gaps. India's COVID-19 vaccination drive involving approximately 1.5 million vaccinators demonstrated that digital transformation at national scale is feasible (41).

### 9.2 Tanzania (genomics and EIR)

Tanzania's TImR, launched in 2017, enabled analysis of migration and dropout patterns from nearly one million patient visits. Tanzania has simultaneously invested in genomic surveillance capacity, with sequencing capacity increasing post-2021, and now mandates digital data sharing across programs in its National Health Research Policy (42).

### 9.3 Kenya (one health)

Kenya's One Health platform integrates human and animal health surveillance for zoonoses such as Rift Valley Fever, combining mobile phone reporting of animal deaths with clinic reports of human febrile illness in a joint database. During a 2020 Rift Valley Fever alert, this system allowed cross-verification of animal outbreaks with human cases, accelerating vaccine ring campaigns in high-risk counties (Moderate evidence from a multicountry East African evaluation demonstrated that integrated One Health surveillance identified zoonotic threats a median of 18 days earlier than conventional human-health surveillance alone). However, operational One Health platforms remain uncommon across most LMICs, limiting generalizability (43).

These examples demonstrate that tailored integration strategies, combining interoperable technology with strong governance can enhance both outbreak response and vaccine delivery. Multi-sector coordination, supported by bodies such as Africa CDC and ASEAN CDC, is a key enabler. Although several digital surveillance platforms have demonstrated substantial improvements in reporting timeliness, vaccine logistics, and immunization coverage, much of the current evidence derives from implementation studies conducted under favorable operational conditions or within well-resourced national programmes. Consequently, the reported effect sizes should be interpreted as demonstrating the potential effectiveness of integrated digital surveillance rather than universally achievable outcomes across all LMIC health systems. Future multicountry prospective evaluations conducted under routine programme conditions are needed to establish the external validity, scalability, and sustainability of these interventions in diverse resource-constrained settings.

## 10 Future directions

We envision surveillance architectures in which environmental sensors, sequencers, point-of-care diagnostics, and community case reports are fed into centralized analytics clouds where machine learning models run continuously to notify health managers of anomalies that can only be identified by multi-source correlation (44). This system's integration of vaccine logistics data would guarantee that the appropriate supply is quickly positioned once a risk is identified. LMICs must keep pursuing the Digital Health Agenda, which includes putting in place national eHealth frameworks, embracing interoperability standards, and making workforce capacity investments, in order to realize this ambition. Instead of short-term pilots, international support should concentrate on sustainable data ecosystems (45, 46).

Importantly, the current evidence base suggests that AI-enabled surveillance should be viewed as an enabling technology rather than a replacement for conventional public health surveillance. While several proof-of-concept studies have demonstrated promising predictive performance, relatively few systems have undergone prospective evaluation under routine LMIC operational conditions. Before widespread implementation, AI models require external validation across diverse epidemiological settings, local calibration to account for population-specific disease patterns, continuous performance monitoring, and governance mechanisms addressing transparency, accountability, privacy, and algorithmic fairness. Accordingly, the greatest near-term value of AI lies in augmenting epidemiological decision-making through timely risk prioritization while preserving expert human oversight for verification and public health response.

## 11 Limitations

As a structured narrative review, this study was designed to synthesize contemporary evidence rather than provide the exhaustive evidence mapping expected of systematic or formal scoping reviews. First, as a structured narrative rather than a systematic review, it cannot provide pooled quantitative estimates; findings are illustrative rather than inferential. Second, the literature search focused predominantly on English-language sources, potentially underrepresenting Francophone Africa, Latin America, and Southeast Asia. Third, most quantitative data derive from intensively supported pilot programmes, limiting generalizability to routine health system contexts. Fourth, publication bias is likely: studies reporting positive outcomes are more frequently published. Fifth, the rapid pace of technological change means some cited systems may be superseded. These limitations underscore the need for rigorous prospective evaluations.

## 12 Conclusions

The path from fragmented data to integrated, collaborative surveillance lies in leveraging digital technologies thoughtfully and equitably. LMICs have begun to chart this course evidenced by the growth of digital vaccine networks and increasing scholarly attention to integrated surveillance but coordinated action is needed to scale successes. While strong evidence supports interoperable digital surveillance platforms and electronic immunization systems, moderate evidence currently supports AI-enabled epidemic intelligence and integrated One Health surveillance, underscoring the need for prospective validation, standardized implementation, and broader evaluation across diverse LMIC settings before widespread adoption.
