# Supplementary material for: From fragmented to integrated surveillance in LMICs: digital pathways for outbreak detection and vaccine intelligence
Source: Front Public Health. 2026 Aug 7;14:1843782. doi: 10.3389/fpubh.2026.1843782 (PMC13493578; doi:10.3389/fpubh.2026.1843782)
Supplement: Supplementary file 1 [file Data_Sheet_1.pdf]

Supplementary Table S1 summarizing the evidence

**Domain**	**Representative study**	**Study design**	**Evidence grade**	**Key finding**
Digital surveillance	Ciecierski-Holmes et al.	Systematic review	**Strong**	Improved surveillance quality
eVIN	Gurnani et al.	National programme evaluation	**Strong**	76% stockout reduction
Tanzania EIR	Mwanza et al.	Observational evaluation	**Moderate**	12.3 percentage-point coverage gain
Kenya SMS	Malhotra et al.	Prospective implementation	**Moderate**	Reporting delay reduced
AI surveillance	Syrowatka et al.	Scoping review	**Moderate**	Limited external validation
One Health	Mariner et al.	Multicountry evaluation	**Moderate**	Earlier outbreak detection

Supplementary Table S2 – Study Design

**Outcome**	**Study type**	**Implementation setting**	**Interpretation**
Reporting delay	Prospective implementation study	Kenya (47 facilities)	Promising, requires wider validation
Stockout reduction	National programme evaluation	India (eVIN)	Strong implementation evidence
Coverage gain	Observational evaluation	Tanzania	Context-specific
Dropout reduction	District pilot	Bangladesh	Preliminary
Cold-chain response	Single-site pilot	Nigeria	Exploratory
